# A new genetic cause of spastic ataxia: the p.Glu415Lys variant in *TUBA4A*

**DOI:** 10.1007/s00415-023-11816-w

**Published:** 2023-07-07

**Authors:** Annalaura Torella, Ivana Ricca, Giulio Piluso, Daniele Galatolo, Giuseppe De Michele, Mariateresa Zanobio, Rosanna Trovato, Giovanna De Michele, Roberta Zeuli, Chiara Pane, Sirio Cocozza, Francesco Saccà, Filippo M. Santorelli, Vincenzo Nigro, Alessandro Filla

**Affiliations:** 1 Department of Neurosciences, Reproductive and Odontostomatological Sciences, Federico II University, Naples, Italy; 2 Department of Precision Medicine, University of Campania, Luigi Vanvitelli, Caserta, Italy; 3 Telethon Institute of Genetics and Medicine, Pozzuoli, Italy; 4 Molecular Medicine, IRCCS Stella Maris Foundation, Pisa, Italy; 5 Institute of Biostructure and Bioimaging, National Council of Research, Naples, Italy

**Keywords:** Tubulinopathy, *TUBA4A*, Spastic ataxia

## Abstract

Tubulinopathies encompass neurodevelopmental disorders caused by mutations in genes encoding for different isotypes of α- and β-tubulins, the structural components of microtubules. Less frequently, mutations in tubulins may underlie neurodegenerative disorders. In the present study, we report two families, one with 11 affected individuals and the other with a single patient, carrying a novel, likely pathogenic, variant (p. Glu415Lys) in the *TUBA4A* gene (NM_006000). The phenotype, not previously described, is that of spastic ataxia. Our findings widen the phenotypic and genetic manifestations of *TUBA4A* variants and add a new type of spastic ataxia to be taken into consideration in the differential diagnosis.

## Introduction

Tubulinopathies encompass a wide overlapping range of brain malformations caused by pathogenic variants of genes encoding for different isotypes of tubulin. Alpha- and β-tubulins are the major components of microtubules, key cytoskeletal components of neurons, where they are essential for cell division, polarity, and intracellular trafficking [1]. Mutations in genes encoding for tubulin subunits (*TUBA1A, TUBB2B, TUBB3, TUBB2A*, *TUBB5,* and *TUBG1*) have been associated with a broad spectrum of neurodevelopmental disorders, usually transmitted in an autosomal dominant manner and mostly characterized by non-progressive complex brain malformations. More than 95% of patients diagnosed with a tubulinopathy have a de novo pathogenic variant [2]. Although tubulinopathies usually include neurodevelopmental disorders, mutations in *TUBB2A* [3] and *TUBA4A* [4] may also underlie neurodegenerative disorders.

*TUBA4A* encodes for α-tubulin, which polymerizes with β-tubulin to form the microtubule cytoskeleton. Twelve non-synonymous variants in *TUBA4A* and four changes leading to premature truncation were identified in about 1% of familial amyotrophic lateral sclerosis (ALS), and 0.4% of sporadic ALS patients [4–6]. Since there is no evidence of *TUBA4A* variants co-segregating with ALS in the affected families, a causal effect of *TUBA4A* in the disease is insufficiently demonstrated, according to Nguyen [7]. Although ALS is the predominant phenotype in *TUBA4A* mutation carriers, a few cases were diagnosed with cognitive problems or frontotemporal dementia (FTD) with or without ALS [4; 6; 8–9], and one case showed nigropathy with parkinsonism without ALS [10].

In this paper, we present a multigenerational family with 11 affected individuals and an unrelated sporadic case, all with an unreported phenotype characterized by cerebellar and pyramidal signs associated with a novel missense mutation falling in the C-terminal domain of the α-tubulin subunit.

## Patients and methods

**Fig. 1:**
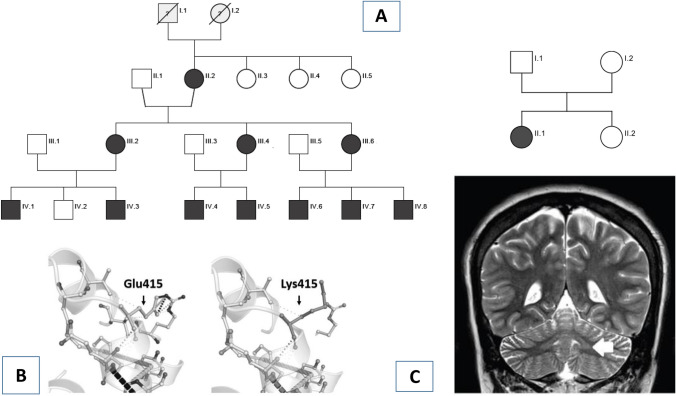
**A** Pedigrees of Families 1 and 2. **B** In silico evaluations of the missense variant p.Glu415Lys identified in the two families. Data suggest that the mutation is harmful to normal protein function. TUBA4A protein modeling of the novel mutation was carried out using the HOPE webserver (https://www3.cmbi.umcn.nl/hope/), which analyzes structural effects of missense mutations retrieving information related to the amino acid sequence and a calculation of the three-dimensional protein structure (using modeling webtools as in [21]). The side chains of wild-type and mutated residues are in dark gray and are also represented as sticks alongside the surrounding residues, which are involved in any type of interaction. Calculation of differential stabilizing energy using the AlphaFold Protein Structure Database (https://alphafold.ebi.ac.uk) shows that the replacement of lysine for glutamic acid at residue 415 likely leads to a destabilizing effect. **C** Patient IV.5, age 29 years: Coronal T2-weighted MRI showing hyperintensity of the dentate (white arrow) and possible enlargement of the interfolial spaces in the crus I–II

Family 1 (F1) is from southern Italy and belongs to a series of 116 families with dominant ataxias present in the University of Naples “Federico II” Ataxia Center database. Family 2 (F2) is from a European research project investigating 340 kindreds with hereditary ataxias and spastic paraplegias by WES/WGS, collected from the IRCCS Stella Maris in Pisa. F1 includes 11 affected members in three generations, while a single patient is present in F2 (Fig. 1A). AF examined all patients from F1, and IR examined the F2 patient. All patients were evaluated according to the Scale for Assessment and Rating of Ataxia (SARA: score 0 = normal; 40 = most severe ataxia) [11]. Stages were defined according to the Inherited Ataxia Progression Scale (IAPS: Stage 1, asymptomatic patient; Stage 2, independent walking; Stage 3, loss of independent walking; Stage 4, wheelchair bound) [12].

Brain MRI was performed in all patients belonging to generation II and III, and in four patients in generation IV in F1 (IV-1; IV-3; IV-4; IV-5), and in the patient from F2. Electromyography (EMG; F1: II-2; IV-4; F2: II-1), peripheral nerve conduction study (PNCS; F1: II-2; III-2; III-4; IV-4; IV-5; F2: II-1), motor evoked potentials (MEP; F1: II-2; III-2; III-4; IV-4; IV-5; F2: II-1), somatosensory evoked potentials (SSEP; F1: II-2; III-2; III-4; III-6; IV-5), brainstem auditory evoked potentials (BAEP; F1: II-3; III-2; III3; III-6) and visual evoked potentials (VEP F1: II-3; III-2; III3; III-6) were performed in selected patients. Cognitive function was evaluated clinically in all individuals and using the mini-mental state examination (MMSE) in four patients (F1: III-4; III-6; IV-4; IV-5).

### Molecular analyses

Blood samples were obtained after informed consent from all affected individuals in both families, from three unaffected individuals in F1 (II-3; II-4; IV-2), and from the unaffected parents of the proband in F2. Pathological expansions of CAG repeats in SCA1-3, SCA6-7, SCA12, and SCA17, of ATTCT in SCA10, and of GAA in SCA27B genes were excluded in index cases of the two families.

### Family 1

Affected members from F1 (II-2; III-2; III-4; IV-4; IV-5; IV-6; IV-7; IV-8) were analyzed by whole exome sequencing (WES). For library preparation of single samples, we used SureSelect QXT Clinical Research exome v2 and Human All Exon V7 kits compatible with Illumina platform version F0, August 2020 (Agilent Technologies, Santa Clara, CA, USA) following the manufacturer’s instructions. Enriched DNA was validated and quantified using a High Sensitivity DNA kit and TapeStation Analysis Software v3.2 (Agilent Technologies). The libraries were sequenced using a NovaSeq6000 system (Illumina, San Diego, CA, USA) by performing paired-end runs covering at least 2X150 nt. The generated sequences were analyzed using an in-house pipeline designed to automate the analysis workflow, as previously described [13]. Variant filtering was carried out by selecting from the database non-synonymous single nucleotide variations (SNVs) and insertions-deletions (indels), with a minor allele frequency (MAF) < 1%. Successive variant filtering was performed based on the absence of selected SNVs and indels in unrelated database samples and on the conservation of both types of variations, leading to a final selection of rare, possibly causative, variations. The transcript NM_006000 (*TUBA4A*) was used for variant annotation. Other routinely used in silico tools such as Varsome 30, PolyPhen-2 31, and SIFT 32 were also exploited to classify the variants. GnomAD and dbSNPs databases were used to assess population frequency.

#### Sanger sequencing

Direct Sanger sequencing using specific primers was performed with a BigDye v3.1 sequencing kit (Applied Biosystems, Waltham, MA, USA) on a 3500xl Genetic Analyzer (Applied Biosystems).

The sequence primers used to validate the *TUBA4A* variant in family 1 were TUBA4A_ E415K: Forward 5’- GAGATCACCAATGCCTGCTTTG -3’, TUBA4A_ E415K; Reverse 5’-ACTCAGAGGGAACAAGAAACCG-3’.

### Family 2

An NGS panel including 285 genes responsible for ataxia and another panel of 142 genes associated with ALS and spastic paraplegia had been run, prior to this study, in F2:II-1 as described elsewhere [14]. Family trios and Sanger sequencing segregation studies were performed as previously described [15].

## Results

### Family 1

The mean age at onset ± SD was 21.6 ± 6.5 years (11–30). The mean disease duration was 13.0 ± 11.8 (0–34). One patient was wheelchair bound at the age of 54 years after 29 years of disease. Three further patients needed support at a mean age of 40.0 ± 3.0 after 12.0 ± 2.6 years of disease. The remaining patients walked independently. The mean SARA score was 9.4 ± 8.3, and SARA progression (defined as the difference between SARA last  visit-SARA first visit/years of  follow-up) was 0.7 ± 0.3 (in nine patients, for whom follow-up was available). The median IAPS stage was 2 (1–4).

Gait disturbance was a constant sign at the onset. Nystagmus, saccadic smooth pursuit, increased knee jerks, lower limb tonus, and Babinski signs were constant. Dysmetria, lower limb weakness, and bladder disturbances were frequent. Decreased vibration sense was present in some patients. Cognition and behavior were always normal. Dysphagia, fasciculations, cramps, and extrapyramidal features were absent, although tremor was present in one individual (Table 1). Four tested patients had mean ± SD MMSE 29.2 ± 0.5. Brain MRI showed slight cerebellar atrophy in seven cases and was normal in one with a short disease duration. Dentate nuclei hyperintensities in T2-weighted sequences were present in the five patients belonging to generations III and IV that were available for re-evaluation (Fig. 1C). EMG, performed in two patients from F1 with disease duration of 11 and 33 years, was normal. PNCS, performed in five patients, showed slight sensory and motor abnormalities in two (IV-4; IV-5). MEP and SSEP were always abnormal with higher involvement at lower limbs. In contrast, VEP and BAEP were always normal.

**Table 1:** Clinical features

	Family 1											Family 2
Patient	II-2	III-2	III-4	III-6	IV-1	IV-3	IV-4	IV-5	IV-6	IV-7	IV-8	II-1
Sex	F	F	F	F	M	M	M	M	M	M	M	F
Age at onset (years)	26	30	26	28	22	15	11	12	25	24	19	DMD
DD (years)	34	12	29	18	6	7	21	17	0	0	0	NA
IAPS stage	4	3	3,5	3,5	2	2	2	2	1	1	1	3
SARA score	24	13	21	18	5	2	6	7	5	2	0	19
SARA progression	0,7	1,0	0,7	1	0,8	0,3	0,3	0,4	NA	NA	NA	0.5
DD to walking aids (years)	NA	10	11	15	–	–	–	–	–	–	–	15
DD to wheelchair (years)	29	–	–	–	–	–	–	–	–	–	–	–
Gait	+++	++	++	++	+	+	+	+	+	+	+	++
Nystagmus	+	+	+	+	+	+	+	+	+	+	+	+
BSP	+	+	+	+	+	+	+	+	+	+	+	+
Slow saccades	+	–	–	–	–	–	–	–	–	–	–	–
Dysarthria	+	+	+	+	+	–	+	+	+	+	–	+
Dysmetria	+	+	+	+	+	–	–	–	+	–	–	±
Dysphagia	–	–	–	–	–	–	–	–	–	–	–	±
Brisk knee jerks	+	+	+	+	++	++	++	++	+	+	+	+
Ankle clonus	+	+	+	+	+	–	+	++	–	++	++	–
Upper limb increased tonus	–	–	–	–	–	–	–	–	–	+	–	–
Lower limb increased tonus	+	+	+	+	+	+	+	+	+	+	+	+
Lower limb weakness	+	+	+	+	–	–	–	+	–	–	–	+
Babinski signs	+	+	+	+	+	+	+	+	+	+	+	+
Fasciculations/cramps	–	–	–	–	–	–	–	–	–	–	–	–
Decreased vibration	±	+	–	–	–	–	–	–	+	–	–	+
Bladder	+++	+	+	++	–	–	–	+	–	–	–	–
Cognitive	–	–	–	–	–	–	–	–	–	–	–	DYS
Behav/psy	–	–	–	–	–	–	–	–	–	–	–	–
MMSE		NP	29	29	NP	NP	30	29	NP	NP	NP	NP
Cerebellar atrophy at MRI	±	+	+	+	+	-	±	±	NP	NP	NP	+
PNCS	±	–	–	NP	NP	NP	±	±	NP	NP	NP	+
EMG	–	NP	NP	NP	NP	NP	–	NP	NP	NP	NP	+
MEP	NP	+	+	+	NP	NP	+	+	NP	NP	NP	+
SSEP	+ +	+	+	+	NP	NP	+	+	NP	NP	NP	NP
BAEP	–	–	–	–	NP	NP	NP	NP	NP	NP	NP	NP
VEP	–	–	–	–	NP	NP	NP	NP	NP	NP	NP	NP

*SARA* (Scale for Assessment and Rating Ataxia [11]) progression:SARA last visit-SARA first visit/ follow-up yrs; *IAPS* (Inherited Ataxia Progression Scale [12]); *DD* disease duration; *DMD* delayed motor development; *BSP* broken smooth pursuit; *DYS* dysexecutive syndrome; *NP*  not performed; *NA*  not assessable;–  absent/normal; severity =  + to +++ ; tendon reflexes ++  = clonus; bladder symptoms +++  = incontinence; *PNCS*  peripheral nerve conduction study; *EMG* electromyography; *MEP* motor evoked potentials; *SSEP* somatosensory evoked potentials; *BAEP* brainstem evoked potentials; *VEP* visual evoked potentials

### Family 2

The patient is a 45 year-old woman born to healthy, non-consanguineous parents. Family history was negative for neurological disorders. Motor development was delayed with independent walking achieved at 24 months; she was always clumsy. The patient graduated from high school and attended one year of postgraduate school (14 years of education). Spasticity became evident at 15 years. She lost independent walking by the age of 30 years. She was treated with botulin toxin and underwent elongation of Achilles’ tendons. The neurological examination is reported in Table 1. The cognitive assessment showed marked dysexecutive syndrome. Brain MRI showed questionable upper vermis atrophy. PNCS, performed at 40 years, showed an axonal sensory-motor neuropathy and EMG signs of chronic denervation without signs of acute denervation at the lower limbs. MEP were abnormal in the upper and lower limbs.

### Molecular analyses

#### Family 1

WES, variant analysis, and prioritization were performed on eight affected members in F1. Variants predicted to be deleterious were prioritized based on the functional relevance of genes, taking into account X-linked, autosomal dominant, and autosomal recessive inheritance models, allowing the identification of a missense variant (c.1243G > A; p.Glu415Lys) in *TUBA4A* as the only strong candidate. Sanger sequencing validated this variant in all the WES patients and in three further affected individuals. The three unaffected family members available for analysis did not carry the variant. The novel p.Glu415Lys variant is not present in gnomAD database and is classified as likely pathogenic according to the American College of Medical Genetics (ACMG) guidelines [16]. The mutation is deleterious (CADD PHRED score 24.4; Revel score 0.82) and involved a residue highly conserved through evolution from human to zebrafish localized in the conserved tubulin C-terminal protein domain (IPR023123). Using freely available tools to assess the impact of missense variants in protein stability and assembly [17], we showed that the p.Glu415Lys variant had a likely destabilizing effect (ΔΔG − 0.61) on α-tubulin stability (Fig. 1B).

### Family 2

Trio-WES showed a heterozygous variant (c.1243G > A; p.Glu415Lys) in *TUBA4A* in the index patient. Analysis of parental DNA showed that the variant is de novo. False paternity was excluded by WES (data not shown).

## Discussion

In the present study, we report two families carrying a novel heterozygous missense mutation, p.Glu415Lys, in the *TUBA4A* gene, resulting in a previously unreported progressive neurological disorder characterized by signs of impairment of upper motor neuron and cerebellum. Brain MRI showed slight or absent cerebellar atrophy. The clinical picture is different from previous phenotypes associated with *TUBA4A* mutations that encompass motor neuron disease with signs of first and second motor neurons (ALS-like), FTD or non-specific dementia with or without ALS, and parkinsonism without ALS in one case [4–6, 8–10]. The p.Glu415Lys mutation caused clinical and laboratory signs of impairment only of the upper motor neuron in F1. The signs of isolated chronic denervation at EMG (not typical for ALS), the delayed motor development (suggesting an earlier onset), and the marked dysexecutive syndrome differentiate the F2 patient from F1 affected members. Notably, the presence of cerebellar impairment has not been previously associated with *TUBA4A* variants. Cerebellar involvement appears to be modest at an early stage and mainly consists of abnormal ocular movements (nystagmus, broken smooth pursuit), and sometimes dysarthria.

A correlation between genotype and phenotype has been proposed with mutations localized in C-terminus being linked to ALS phenotype and variants localized in N-terminus associated with FTD [8]. In our case, the novel mutation localized in the C-terminus domain is associated with a spastic ataxia phenotype. The mechanism by which p.Glu415Lys leads to these clinical manifestations is unclear. It might be related to the inability of mutant *TUBA4A* to form microtubules by impacting on α/β-tubulin dimerization. Alternatively, mutant *TUBA4A* could affect motor kinesin domains. A similar mechanism has been proposed for the p.Asp417Asn variant in the neuron-specific β-tubulin *TUBB2A*, also leading to spastic ataxia [3]. However, we cannot exclude that additional and as yet unidentified variants in inherited ataxia or spastic paraplegia genes may modify the clinical presentation.

Thus, the p.Glu415Lys variant in *TUBA4A* extends the list of spastic ataxias that encompass both dominant (polyQ and non-polyQ spinocerebellar ataxias; SCAs) or recessive forms (Table 2) [18, 19]. In polyQ SCAs, the presence of a pyramidal syndrome is linked to the size of the CAG repeat expansion [20]. This frequent co-occurrence of ataxia and spasticity may be driven by the shared vulnerability of corticospinal tracts and cerebellar circuits.

**Table 2:** Spastic Ataxias

Dominant Spinocerebellar Ataxias, SCAs	Recessive
Disease/Gene	
PolyQ SCAs	Ataxia at forefront
SCA1	Friedreich’s ataxia/*FXN*
SCA3	SPAX6 (ARSACS)/*SACS*
SCA7	CTX/*CYP27A*
Non-PolyQ SCAs	*NPC*
*CACNA1A*	*POLR3A*
SCA5/*SPTB2*	*POLR3B*
SCA13/*KCN3*	SCAR1 (AOA2)/*SETX*
SCA14/*PRKCG*	SCAR16/*STUB1*
SCA15/*ITPR1*	SCAR18/*GRID2*
SCA28/*AFG3L2*	Spasticity at forefront
SCA48/*STUB1*	*SPG7*
	SPAX5 (SCA28)/*AFG3L2*
	SPG39/*PNPLA6*
	SPG46/*GBA2*
	SPG76/*CAPN1*
	*FOLR1*
	*TUBB2A*

## Data Availability

The data that support the findings of this study are available on reasonable request from the corresponding author.
